# Metabolic Engineering of Cofactor F_420_ Production in *Mycobacterium smegmatis*

**DOI:** 10.1371/journal.pone.0015803

**Published:** 2010-12-29

**Authors:** Ghader Bashiri, Aisyah M. Rehan, David R. Greenwood, James M. J. Dickson, Edward N. Baker

**Affiliations:** 1 Structural Biology Laboratory, School of Biological Sciences, The University of Auckland, Auckland, New Zealand; 2 Maurice Wilkins Centre for Molecular Biodiscovery, School of Biological Sciences, The University of Auckland, Auckland, New Zealand; 3 Centre for Genomics and Proteomics, School of Biological Sciences, The University of Auckland, Auckland, New Zealand; National Institute for Medical Research, Medical Research Council, United Kingdom

## Abstract

Cofactor F_420_ is a unique electron carrier in a number of microorganisms including Archaea and Mycobacteria. It has been shown that F_420_ has a direct and important role in archaeal energy metabolism whereas the role of F_420_ in mycobacterial metabolism has only begun to be uncovered in the last few years. It has been suggested that cofactor F_420_ has a role in the pathogenesis of *M. tuberculosis*, the causative agent of tuberculosis. In the absence of a commercial source for F_420_, *M. smegmatis* has previously been used to provide this cofactor for studies of the F_420_-dependent proteins from mycobacterial species. Three proteins have been shown to be involved in the F_420_ biosynthesis in Mycobacteria and three other proteins have been demonstrated to be involved in F_420_ metabolism. Here we report the over-expression of all of these proteins in *M. smegmatis* and testing of their importance for F_420_ production. The results indicate that co–expression of the F_420_ biosynthetic proteins can give rise to a much higher F_420_ production level. This was achieved by designing and preparing a new T7 promoter–based co-expression shuttle vector. A combination of co–expression of the F_420_ biosynthetic proteins and fine-tuning of the culture media has enabled us to achieve F_420_ production levels of up to 10 times higher compared with the wild type *M. smegmatis* strain. The high levels of the F_420_ produced in this study provide a suitable source of this cofactor for studies of F_420_-dependent proteins from other microorganisms and for possible biotechnological applications.

## Introduction

**Figure 1:**
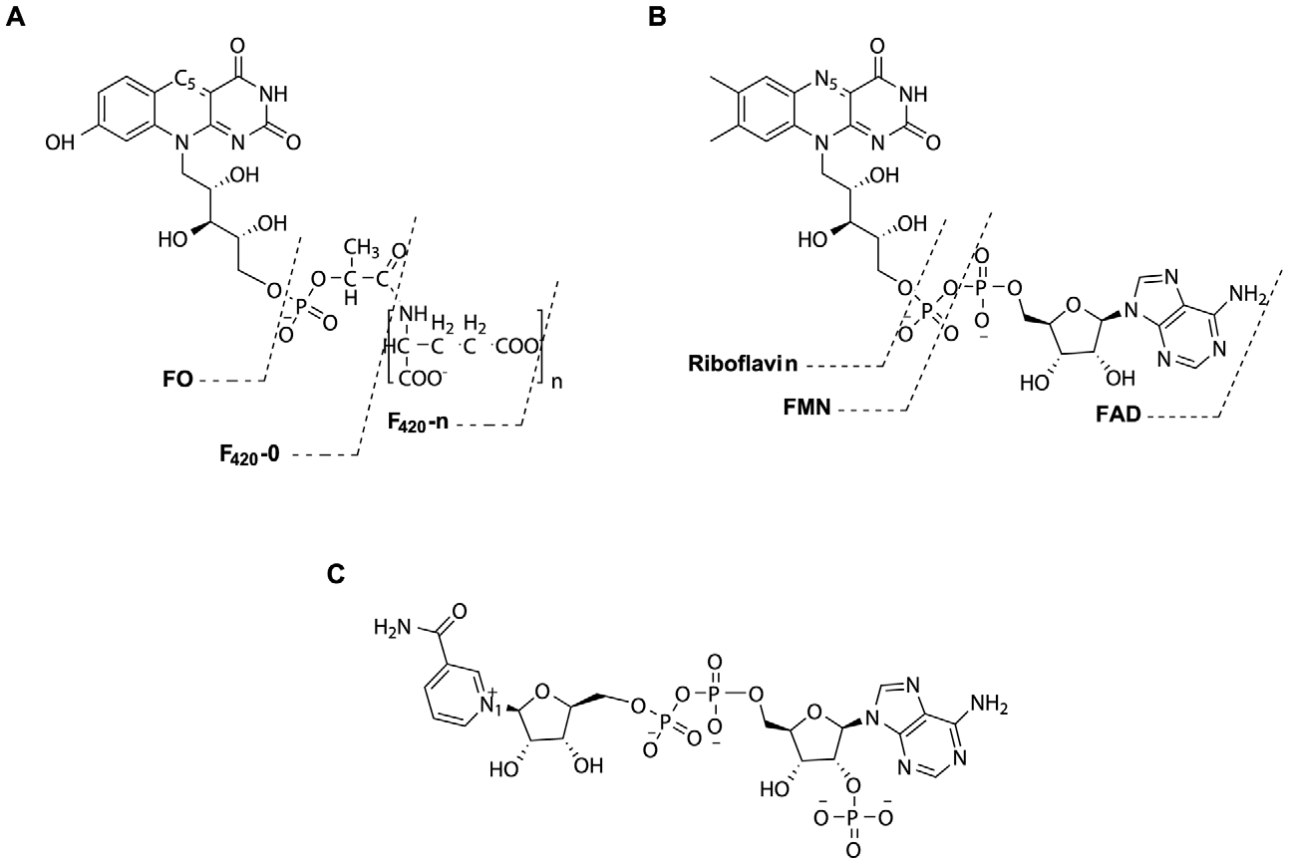
The molecular structures of F_420_, the flavins and NADP^+^. (A) Schematic representation of F_420_ showing different parts of the molecule, whereas (B) and (C) show the molecular structures of the flavins and NADP^+^, respectively. The atoms involved in oxidoreductive reactions are numbered in all structures.

The cofactor F_420_ was first identified chemically in methanogenic Archaea in 1972 [1], although a compound with similar characteristics was previously described in Mycobacteria in the early 1960s [2], [3]. Since its discovery, F_420_ and its precursor FO (so called 5-deazaflavins) have been found in a variety of (micro)organisms, including Archaea, bacteria and eukaryotic species (Table 1). F_420_ is named on the basis of its intense absorbance/fluorescence at 420 nm (emission 480 nm), which is redox dependent and is lost upon reduction of the cofactor. It also has unique chemical and biological characteristics; the isoalloxazine chromophore of F_420_ is structurally very similar to that of the flavins (FMN and FAD), although it is functionally similar to NAD(P)^+^ (Figure 1). Functionally, F_420_ is a two–electron carrier involved in hydride transfer reactions. The redox potential of F_420_H_2_/F_420_+2e^−^ (−360 mV) is lower than those of the classical hydrogen carriers NAD(P)H/NAD(P)+2e^−^ (−320 mV) and FADH_2_/FAD+2e^−^ (−219 mV) [4], [5].

**Table 1:** Deazaflavin–dependent reactions in different (micro)organisms.

Organism	Enzyme/Activity	Function	Reference
Archaea	F_420_ hydrogenase	Energy metabolism, Oxygen and sulfite detoxification, Oxygen sensing	[40]
	F_420_H_2_ dehydrogenase		[41]
	F_420_H_2_ oxidase		[42]
	F_420_H_2_:NADP^+^ oxidoreductase		[43]
	F_420_H_2_:heterodisulfide oxidoreductase		[44]
	F_420_H_2_:quinone oxidoreductase		[45]
	F_420_:formate dehydrogenase		[46]
	F_420_:Pyruvate synthase		[47]
	F_420_:α-ketoglutarate synthase		[48]
	Methylenetetrahydromethanopterin dehydrogenase		[49]
	Methylenetetrahydromethanopterin reductase		[50]
	Secondary alcohol dehydrogenase		[51]
	Sulfite reductase		[52]
	F_390_ synthetase		[53]
	F_390_ hydrolase		[54]
Actinobacteria	Tetracycline and Lincomycin synthesis	Antibiotic biosynthesis	[55], [56], [57]
	F_420_:NADPH oxidoreductase and F_420_ hydride transferase	Biodegradation of nitrophenol compounds	[58]
	Glucose-6-phosphate dehydrogenase	Providing F_420_H_2_ inside cells	[14]
	Deazaflavin–dependent nitroreductase	PA–824 prodrug activation	[15]
	F_420_H_2_–dependent reductases	Xenobiotic metabolism	[25]
Archaea, bacteria and eukaryotes	DNA photolyase	DNA repair and maintenance	[59], [60], [61], [62]

**Figure 2:**
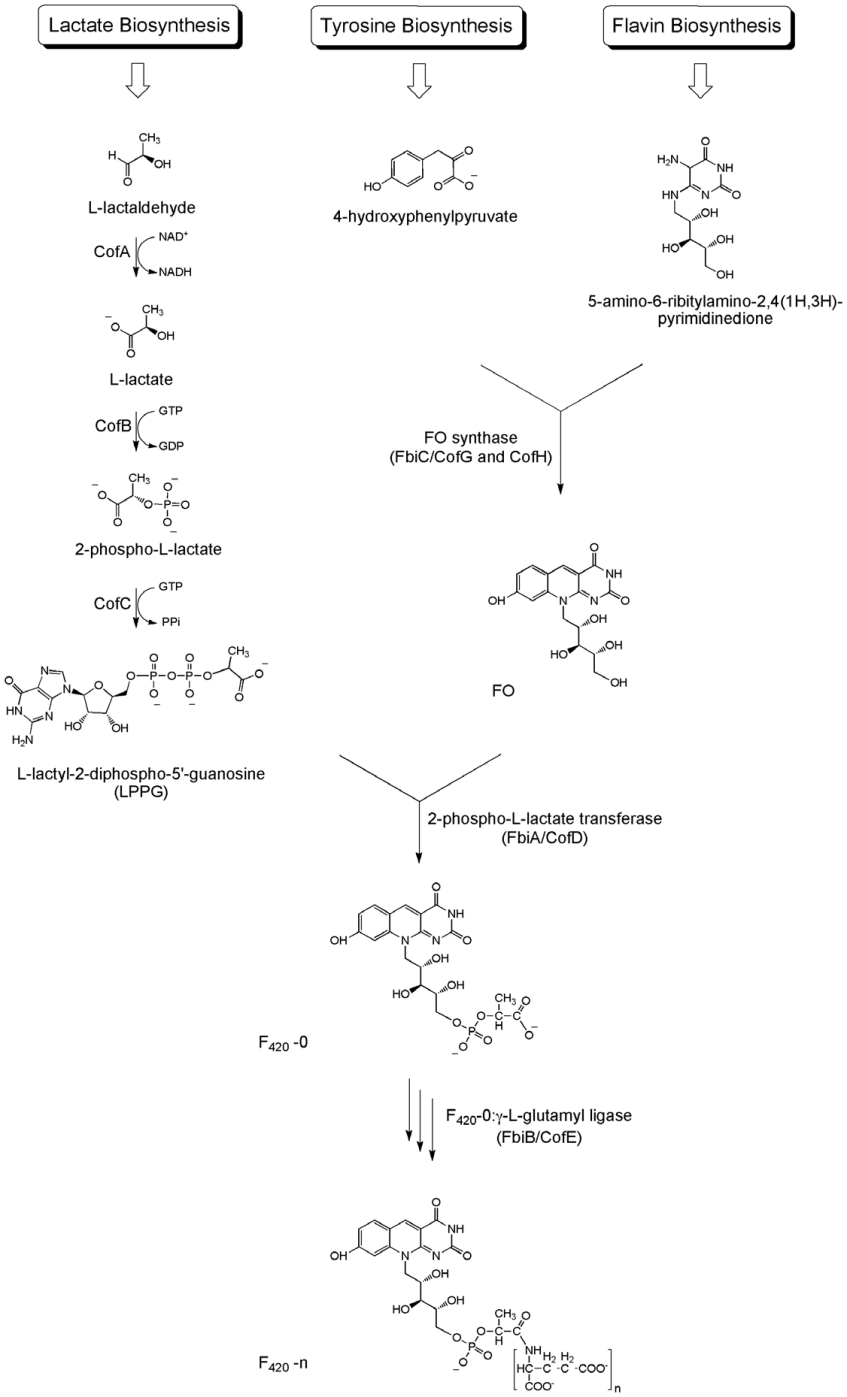
The proposed biosynthetic pathway for cofactor F_420_. The FbiA, B and C are Mycobacterial proteins whereas the CofA, B, C, D, E, G and H are Archaeal proteins involved in the biosynthetic reactions. The pathway to formation of the activated phospholactate moiety (LPPG) is yet to be experimentally established in Mycobacteria. In some Archaeal species an α–linked terminal glutamate residue caps the γ–linked poly–glutamate tail, the addition of which is catalyzed by CofF.

A key biosynthetic precursor of F_420_ is FO (7,8-didemethyl-8-hydroxy-5-deazariboflavin), comprising an isoalloxazine ring and ribitol moieties. Formation of F_420_ follows a series of biochemical reactions and is completed by the addition of a phospholactate group, and finally a poly–glutamate tail in which L–glutamate residues are linked together via γ–glutamyl bonds (Figure 2) [6], [7]. The length of the poly–glutamate tail constitutes the main difference between the F_420_ cofactors from different microorganisms, the number of residues varying from 2–9. There are suggestions, however, that the type of α– or γ–glutamyl linkage in the terminal glutamate residue could also be different in some Archaeal species [6], [8], [9], [10].

F_420_ is not commercially available and researchers working on F_420_–dependent proteins have to prepare it as required. With the discovery of new F_420_–dependent enzymes and increasing interest in F_420_–dependent reactions, especially in the case of the pathogen *Mycobacterium tuberculosis* (Mtb), a resource with high yields of F_420_ production is required. F_420_ has been previously purified from various microorganisms, including Archaea (Methanobacterium, Methanococcus and Methanosarcina species) and Actinomycetes (Actinomadura, Actinoplanes, Streptomyces, Rhodococcus, Nocardia and Mycobacteria species), with differing yields [11]. F_420_ purification in all cases, however, essentially follows the same principle; precipitation of cellular proteins using heat or an organic solvent, followed by separation of F_420_ from remaining cellular components based on its acidic nature [4]. In order to purify F_420_, a number of different chromatographic steps have been used, including ion exchange, adsorption, HPLC and gel filtration chromatography [6], [9], [11]. Isabelle *et al.* have reported thorough analyses of F_420_–producing microorganisms, and based on “ease of growth, fewer hazards, and lower costs” concluded that *M. smegmatis* is the best source for F_420_ production, providing there is no requirement for a particular number of glutamate residues in the F_420_ poly–glutamate tail [11].

Our initial F_420_ purification trials indicated that *M. smegmatis* transformed to over–express the *M. tuberculosis* protein FGD1 (F_420_–dependent glucose-6-phosphate dehydrogenase 1) could produce higher levels of F_420_ compared with the wild type strains. This observation prompted us to thoroughly investigate the effects on F_420_ production of over–expression of other proteins known to be involved in F_420_ biosynthesis and metabolism in Mycobacteria. These include three proteins in the F_420_ biosynthetic pathway, *viz*. FbiA (Rv3261) [12], FbiB (Rv3262) [12] and FbiC (Rv1173) [13] and three other proteins which are shown to be involved in F_420_ metabolism: FGD1 (Rv0407) [9], [14], Ddn (Rv3547) [15] and Rv0132c (author's unpublished data).

Here we describe the development of vectors to co–express Mtb proteins in *M. smegmatis*. We further show that by co–expressing enzymes associated with F_420_ production and manipulating growth conditions, greatly increased levels of F_420_ can be obtained. With the growing recognition that F_420_ plays a crucial role in Mycobacteria and other organisms, this readily available source of the cofactor will be useful for testing its physiological and biochemical roles, and for possible applications in biotechnology.

## Materials and Methods

### Preparation of New Mycobacterial Vectors

The pYUB1049 vector (5795 bp) is a product of ligation between the vectors pMS134 and pET28b–cmaA2 [16], resulting in a vector with a cloned gene between *Nde*I and *BamH*I restriction sites. The pYUB1049 vector was subjected to restriction digestion using *Nco*I (single site) and *Blp*I (two sites) restriction sites, in order to obtain a linear vector without the multiple cloning site. The plasmid was first digested to completion with *Nco*I (Roche Applied Science) and dephosphorylated using calf intestinal alkaline phosphatase (New England Biolabs) followed by ethanol precipitation. The *Nco*I–cut linear pYUB1049 vector was subjected to a partial digestion with *Blp*I (*Bpu*I102I isoschizomer, Fermentas) for 20 minutes and the reactions stopped using 5 µL 0.5 M EDTA. The digested vector was run on a 0.5% agarose gel and a DNA fragment corresponding to 4705 bp was excised and gel–purified.

The pET28b and pETDuet–1 vectors (Novagen) were double–digested using *Nco*I and *Blp*I enzymes. The resulting multiple cloning site fragments, 216 and 382 bp respectively, were ligated separately into the *Nco*I/*Blp*I fragment of the pYUB1049 vector using T4 DNA ligase (Roche Applied Science). Ligation mixtures were electroporated into *E. coli* TOP10 cells and the positive colonies were selected on low salt LB agar plates (tryptone 10 g/L, yeast extract 5 g/L, NaCl 5 g/L and agar 15 g/L, pH 8.0) containing 50 µg/mL hygromycin B. Positive clones were verified using restriction digestion and sequencing. The resulting vectors were designated as pYUB28b (4921 bp) and pYUBDuet (5087 bp), respectively.

### PCR Amplification and Cloning

The open reading frames (ORFs) encoding Rv3261 (FbiA), Rv3262 (FbiB), Rv1173 (FbiC), Rv0407 (FGD1), Rv3547 (Ddn) and Rv0132c were amplified from *M. tuberculosis* H37Rv genomic DNA using *Pwo*, *Pfx* or PrimeStar polymerases with the primers outlined in Table 2. All constructs were cloned with either N– or C–terminal His_6_–tags. The amplified products for the FGD1 [17] and Rv0132c constructs were cloned using restriction/ligation cloning into the pYUB1049/pYUB28b vectors. The constructs were transformed into *E. coli* Top10 cells and plated on low salt LB agar medium supplemented with 50 µg/mL hygromycin B to select for colonies harbouring the plasmid. Positive clones were verified using colony PCR, restriction digestion and sequencing.

**Table 2:** Oligonucleotide primers used in the amplification of the protein coding sequences in this study.

Construct	Primer Sequences (5′–3′)		Restriction Enzyme
Rv3261 (FbiA)	Forward	GGCAGCGGCGCGGTGAAGGTCACCGTTCTG	n.a.
	Reverse	GAAAGCTGGGTGTCAAGCTACCACTCCCGCAAG	n.a.
Rv3262 (FbiB)	Forward	GGCAGCGGCGCGTTGACCGGCCCCGAACATGGC	n.a.
	Reverse	GAAAGCTGGGTGTCACTTCAGGATCAGCAAATC	n.a.
Rv1173 (FbiC–pDESTsmg)	Forward	GGCAGCGGCGCGGTGCCGCAGCCTGTAGG	n.a.
	Reverse	GAAAGCTGGGTGCTAGGCCGCAAGCAGGGC	n.a.
FbiAB	Forward	GTACTGTCCTGC**CATATG**AAGGTCACCGTTC	NdeI
	Reverse	GCCTATCGTC**GATATC**GCGAATGTCAC	EcoRV
Rv1173 (FbiC–pYUBDuet)	Forward	AGGTATACCA**CCATGG**CGCCGCAGCCTG	NcoI
	Reverse	CCCGGCATCG**AAGCTT**GGGCTAGGC	HindIII
Rv3547 (Ddn)	Forward	GGCAGCGGCGCGATGCCGAAATCACCGCCG	n.a.
	Reverse	GAAAGCTGGGTGTCAGGGTTCGCAAACCACGATCGG	n.a.
Rv0132c	Forward	GGGTAGTGCAGC**CCATGG**CCACCGGCATCTCACGGCGG	NcoI
	Reverse	CGTAGGTGC**GGATCC**GCGGCTTCAGCGCAGTTCGGG	BamHI
Gateway Generic Forward		GGGGACAAGTTTGTACAAAAAAGCAGGCTTCGAAAACCTGTATTTTCAGGGCAGCGGCGCG	n.a.
Gateway Generic Reverse		GGGGACCACTTTGTACAAGAAAGCTGGGTG	n.a.

The underlined nucleotides indicate overlapping base pairs with the generic primers for the ORFs cloned using the Gateway® system. The bold underlined nucleotides show the restriction site for the appropriate enzymes, as indicated in the right–hand column.

All other ORFs were cloned using the Gateway® cloning system into the pDESTsmg vector [18]. The Gateway® cloning system uses a nested PCR method involving two rounds of amplification in which the second round uses the product of the first round as template. Gene–specific primers are used in the first round PCR to amplify the gene of interest and generic primers are used in the second round amplification to incorporate the required recombination sites for subsequent cloning. The PCR products were cloned by recombination into pDONR221 (Invitrogen) using BP Clonase™ (Invitrogen), to generate the entry clones. The constructs were transformed into *E. coli* Top10 cells and plated on LB agar medium containing 50 µg/mL of kanamycin. Positive clones were verified using *Bsr*GI digestion and sequencing. These positive entry clones were recombined *in vitro* with pDESTsmg, in an LR reaction using LR Clonase™ (Invitrogen), to generate a *M. smegmatis* expression construct. Following transformation of recombinant pDESTsmg plasmids, positive clones were selected on low salt LB agar plates supplemented with 50 µg/mL hygromycin B and were verified using *Bsr*GI digestion.

The pYUBDuet vector was used to clone the F_420_ biosynthetic ORFs (FbiAB and FbiC) together using restriction/ligation cloning. Both FbiC and FbiAB ORFs were amplified using *PfuUltra* Fusion HS DNA polymerase (Stratagene) using the primers outlined in Table 2. FbiC was first cloned using *Nco*I/*Hind*III restriction sites and the FbiAB operon was subsequently cloned using *Nde*I/*Eco*RV restriction sites.

### Expression in *M. smegmatis*

All expression constructs were electroporated individually into the *M. smegmatis* strain mc^2^4517. Preparation of electrocompetent cells and electroporation procedures were performed following published protocols [19]. Briefly, *M. smegmatis* mc^2^4517 cells were grown at 37°C in 7H9/ADC/Tween80 or LB/Tween80 containing 50 µg/mL kanamycin until an OD600 ∼0.7. Cells were harvested and washed three times in 10% ice–cold glycerol and finally resuspended in 10% ice–cold glycerol. Single aliquots of the resulting competent cells (40 µL) were transformed with 1 µL of DNA and a further 260 µL of 10% glycerol in 0.2 cm cuvettes. Electroporation was performed using a Bio–Rad Gene Pulser set to the following parameters: *R* = 1000 Ω, *Q* = 25 µF and *V* = 2.5 kV. Cells were immediately harvested with 1 mL 7H9/ADC/Tween80 (Difco™ and BBL™ Middlebrook) or LB/Tween80 and incubated for 3 h at 37°C with shaking. Positive transformants were selected by plating on 7H10/ADC (Difco™ and BBL™ Middlebrook) or LBT agar plates containing 50 µg/mL each of kanamycin and hygromycin B.

Protein expression was performed either in autoinduction [20], LB or 7H9/ADC media supplemented with 0.05% Tween80 and 50 µg/mL each of kanamycin and hygromycin B. A single transformed colony was selected from a 7H10/ADC plate and used to inoculate a starter culture in MDG media (25 mM Na_2_HPO_4_, 25 mM KH_2_PO_4_, 50 mM NH_4_Cl, 5 mM Na_2_SO_4_, 2 mM MgSO_4_, 0.5% D-glucose, 0.25% L-aspartate, 0.2× metal mix) [20]. The starter culture was grown for 48–72 h at 37°C and was freshly used at a dilution of 1∶100 to inoculate expression cultures of ZYM–5052 autoinduction (1% tryptone, 0.5% yeast extract, 25 mM Na_2_HPO_4_, 25 mM KH_2_PO_4_, 50 mM NH_4_Cl, 5 mM Na_2_SO_4_, 2 mM MgSO_4_, 0.5% glycerol, 0.05% glucose, 0.2% alpha–lactose, 1× metal mix), LB or 7H9/ADC. The expression cultures were grown for 4 days at 37°C for maximal expression [17]. LB, MDG and 7H9/ADC cultures were induced using IPTG at a final concentration of 0.1 or 1 mM.

### Western Blot Analyses

*M. smegmatis* cells expressing different constructs were lysed twice using a cell disruptor (Constant Systems Ltd.) and centrifuged at 16,000×g to pellet non–lysed cells and other insoluble material. Protein samples were separated on a 15% SDS–PAGE gel and transferred to polyvinylidene difluoride (PVDF) membranes using a wet transfer protocol (200mA, 3 hours) [21]. His–tagged recombinant proteins were detected using a mouse monoclonal anti–His antibody and horseradish peroxidase–conjugated anti–mouse antibody (GE Healthcare). The Luminol (ECL plus kit, GE Healthcare) chemiluminescence was detected using an LAS4000 imaging system (Fujifilm).

### FO and F_420_ Characterization

*M. smegmatis* cells expressing different *M. tuberculosis* proteins were grown in identical conditions to late log phase or stationary phase. In all expression cultures the ZYP–5052 autoinduction media was used for F_420_ production experiments and the media to flask volume ratio was kept constant at 20%. In order to optimize the media for F_420_ production, the ZY component of ZYM–5052 media was replaced by commonly used media bases including 2× ZY, YT (0.8% tryptone, 0.5% yeast extract and 42.77 mM NaCl), TB (1.2% tryptone, 2.4% yeast extract and 0.4% glycerol), SOB (2% tryptone, 0.5% yeast extract, 8.56 mM NaCl, 2.5 mM KCl and 10 mM MgCl_2_) and SOC (SOB with 20 mM glucose). Iron and sulphur supplements (ferric ammonium citrate, ferric citrate and ferrous sulphate all at 0.1 mg/mL and L–cysteine at 1 mM) were also added to the expression media as a possible requirement for the FbiC enzyme. L–glutamate and manganese chloride (1 mM final concentration) were also added to the expression media to evaluate their necessity for FbiB–mediated F_420_ production [22].

To ascertain the optimum growth period for F_420_ production, eight identical cultures of *M. smegmatis* cells expressing the recombinant FbiABC construct were set up. Each culture had a wild type *M. smegmatis* culture as a control. At 24 h intervals, one culture each of control and recombinant FbiABC–expressing *M. smegmatis* cells were harvested and processed to monitor the F_420_ production level. The procedure was carried out for eight days and the F_420_ production ratio for each day was calculated by dividing the F_420_ fluorescence from FbiABC–expressing cells by fluorescence of the wild type control.

*M. smegmatis* cells were centrifuged for 15 min at 16000×g and the resulting media were used for FO characterization. The cell pellets were washed with 25 mM sodium phosphate buffer, pH 7.0 and were subsequently resuspended in 1 mL of the same buffer per 100 mg of cells (wet weight). The cell suspensions were autoclaved at 121°C for 15 min to break the cells open and were then centrifuged for 15 min at 16000×g. Fluorescence of the media and the extract were monitored using excitation wavelength of 420 nm (405±10 nm filter) and emission wavelength of 480 nm (485±15 nm filter). All fluorescence experiments were performed using an EnVision Multilabel plate reader (Perkin Elmer) in a 96–well plate format and were carried out in triplicate.

The autoclaved cell extracts were further purified using a HiTrap QFF ion exchange column (GE Healthcare) to separate the intracellular FO from the F_420_. The extract was run on the column pre–equilibrated with 25 mM sodium phosphate buffer, pH 7.0 and was subsequently washed with five column volumes of buffer. Two yellow fractions were eluted at 200 and 500 mM NaCl, respectively. The purified fractions were used for mass spectrometry analysis, together with the media from the previous step. The media (1 mL) was treated with an equal volume of cold acetone to precipitate the protein and the solution was then evaporated down to <0.5 mL to drive off the acetone. A mix of water and 5% aqueous methanol with 0.1% formic acid was added to bring the final concentration of methanol to less than 1% (total volume 4 mL). All samples were then applied to a pre–equilibrated Alltech Maxi–Clean 300 mg large pore 100Å C–18 SPE cartridge and washed with 4 mL 5% methanol containing 0.1% formic acid followed by 4 mL 10% methanol. Compounds were eluted with 4 mL 80% methanol containing 5 mM ammonium bicarbonate pH 8.5. Eluates were evaporated under nitrogen and redissolved in 80% methanol and 20 mM ammonium acetate ready for mass spectrometry. Samples were infused at 3 µL/min under negative electrospray conditions into an LTQ–FT mass spectrometer (Thermo Scientific). The ion intensity data were obtained using a source voltage of 2.5 kV and capillary temperature of 225°C. Ions were examined in both the ion trap and ion cyclotron resonance cells, the latter to obtain high resolution (100,000 at *m/z* 400) accurate mass data. This was necessary to confirm the atomic composition of the ions and help deconvolute the contribution of metal ion adducts (Na^+^/K^+^) to the levels of individual poly–glutamate species. Up to four sodium ions were adducted to produce some double charged negative ions.

## Results

### New Mycobacterial Expression Vectors

**Figure 3:**
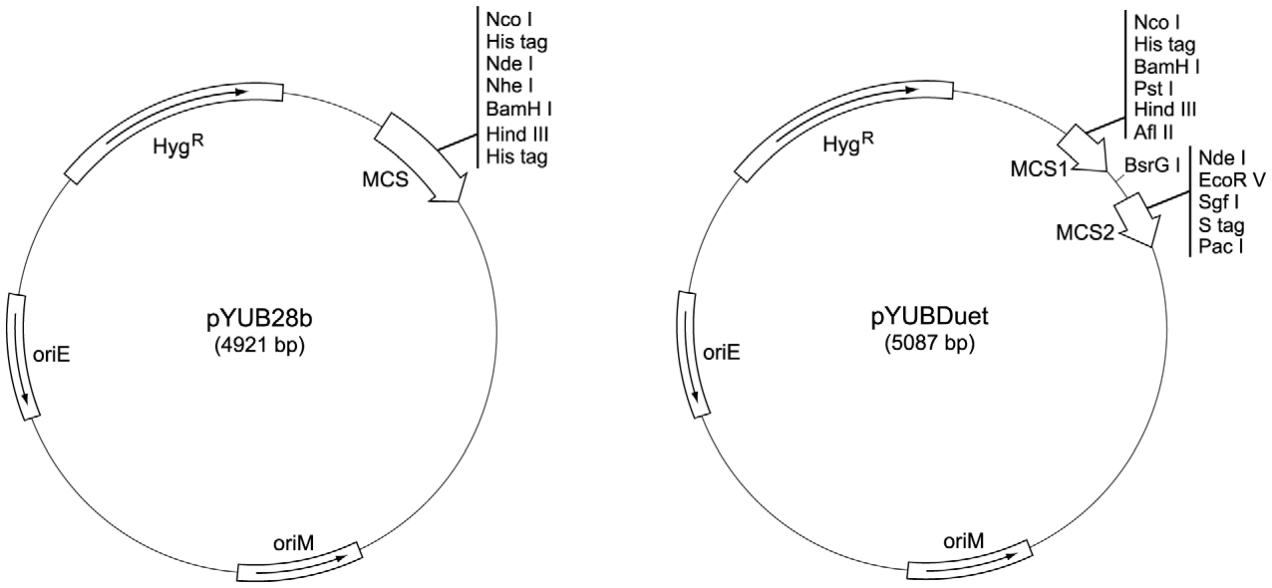
A schematic representation of the vectors designed in this study. The unique restriction sites in the multiple cloning sites of each vector are indicated.

The pYUB1049 vector does not provide an intact multiple cloning site and does not support C–terminal His–tag expression. In order to overcome these obstacles, the pYUB1049 vector was subjected to a restriction digestion using *Nco*I and *Blp*I enzymes and a linear fragment lacking the multiple cloning site was obtained. The resulting fragment was used as a backbone that could be ligated to the intact multiple cloning site from the pET28b or pETDuet–1 vectors to produce the pYUB28b and pYUBDuet vectors, respectively. Figure 3 provides a schematic representation of the vectors with the list of unique restriction sites that can be used for cloning.

### PCR Amplification and Cloning

Six different ORFs which are believed to be involved in F_420_ biosynthesis (FbiA, FbiB and FbiC) or F_420_ metabolism (FGD1, Ddn and Rv0132c) were amplified and cloned for expression in *M. smegmatis* as His–tagged proteins. Assuming that FbiA and FbiB ORFs are transcribed as a single operon, we investigated the possibility of cloning and co–expression of the whole F_420_ biosynthetic pathway (FbiAB and FbiC) in order to boost F_420_ production yield. The pYUBDuet co–expression vector was designed, prepared and subsequently used to clone FbiC and FbiAB ORFs, making it possible to express three different proteins from a single vector. All three proteins were expressed in their native form without His–tags.

### Expression of Proteins in *M. smegmatis*

The six F_420_ biosynthetic or metabolic ORFs cloned into pYUB1049/pYUB28b/pDESTsmg vectors were expressed in *M. smegmatis* as individual proteins. Each of these proteins were cloned with either N– or C–terminal His–tags, making it possible to detect the protein expression using monoclonal anti–His antibodies. The western blotting experiments indicated that all proteins were expressed in *M. smegmatis* cells, as shown by appearance of correct–sized bands for the appropriate proteins (data not shown).

The expression of proteins from the pYUBDuet vector could not be detected using western blotting, as they did not contain any tags; however, their successful expression could be inferred from FO and F_420_ production as discussed later.

### Cofactor F_420_ Production

Individual *M. smegmatis* cultures harbouring six different constructs (FbiA, FbiB, FbiC, FGD1, Ddn and Rv0132c) were grown in order to find out the over–expression effect of these targets on F_420_ production. Three different media were initially used to express the proteins; LBT with IPTG induction, MDG with no or low induction using IPTG, and ZYM–5052 autoinduction media. Based on growth rate and cell mass, ZYM–5052 media was selected as the best media and was used to continue F_420_ production experiments. The fluorescence signals of the expression media and the cell extracts were monitored at 420 nm, enabling the detection of both FO and F_420_. It has been reported that FO comprises 1–7% of the total intracellular deazaflavin in Mycobacteria [8]; we used fluorescence at 420 nm to evaluate the F_420_ contents of the cellular extracts without taking into account the small portion of the fluorescence signal coming from FO.

**Figure 4:**
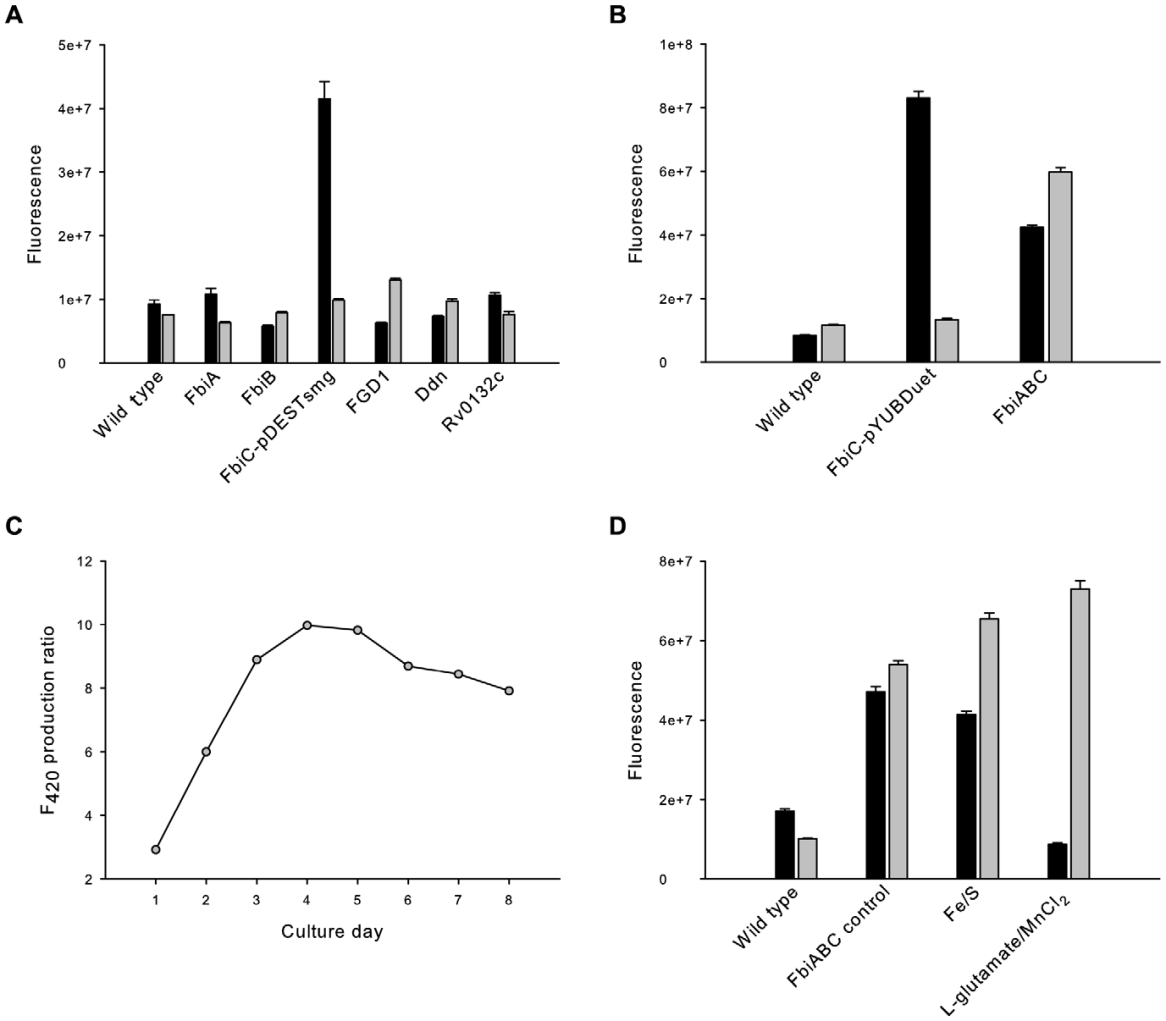
FO/F_420_ production by *M. smegmatis* cells expressing different recombinant proteins. (A) comparative FO/F_420_ production between wild type strain and six different recombinant strains expressing proteins involved in F_420_ biosynthesis or metabolism. (B) FO/F_420_ production using co–expression vector compared to the wild type strain. (C) F_420_ production ratio by *M. smegmatis* cells expressing FbiABC construct over wild type strains for eight days. (D) Effect of iron/sulphur and L–glutamate/manganese additives on the F_420_ production (FO is shown as black and F_420_ as grey). In panels (A), (B) and (D) the error bars are derived from experiments carried out in triplicate.

The experimental results indicate that FGD1 over–expression increases F_420_ production by almost two–fold compared to the wild type strain (Figure 4, A). Cells expressing other Mtb proteins did not show a significant increase in F_420_ yield, however. Cells expressing the FbiC construct (pDEST–FbiC) showed a strong blue–green colour in the media. This is presumably due to the presence of fluorescent FO in the media which diffuses out of the cells as FO does not have any charge on the molecule to cause retention inside the cell (Figure 4, A) [23]. Mass spectrometry confirmed that FO was indeed responsible for the distinct fluorescence of the media (*m/z* 362.09870 [M-H]^−^; C_16_H_16_N_3_O_7_ requires 362.09882). This observation could be explained by over–expression of the FbiC protein leading to higher FO synthesis. Because the cells could not convert the over–produced FO to F_420_, the excess was presumably lost from the cells, either by diffusion or by active export.

This observation provided the motivation for us to co–express the FbiAB operon together with FbiC, hoping that over–expressed FbiA and FbiB proteins would be able to convert the synthesised FO into F_420_ inside the cells. The pYUBDuet vector was used to clone FbiABC ORFs together; FbiC was first cloned, resulting in the pYUBDuet–FbiC construct, after which FbiAB was introduced to obtain pYUBDuet–FbiABC. Both these constructs were used to investigate the effect on FO/F_420_ production (Figure 4, B). Cells expressing FbiC alone (pYUBDuet–FbiC) consistently showed more than 10–fold higher FO levels in the expression media compared to the wild type strains. It is an interesting observation that FO production by the pYUBDuet–FbiC construct is much higher (>50%) than by the pDESTsmg–FbiC construct, with the former expressing FbiC as the native protein whereas the latter has an N–terminal His–tag. In contrast, F_420_ production from pYUBDuet–FbiC was not significantly elevated compared to wild type. By expressing the FbiAB operon together with FbiC (pYUBDuet–FbiABC), however, F_420_ production was consistently more than five times higher inside the cells (Figure 4, B). These results clearly indicate that the cells express functional recombinant proteins resulting in much higher intracellular F_420_ levels.

*M. smegmatis* cells expressing the pYUBDuet–FbiABC construct were then used to find out the optimum time period for F_420_ production. The F_420_ production was monitored for eight days using ZYM–5052 media and the F_420_ production ratio was calculated and plotted versus the day of culture. The results indicated that the F_420_ levels were the highest on day four of the culture, after which the levels gradually decreased. Based on this result, the best time to harvest the cells for F_420_ purification is 4–5 days after setting up the expression culture (Figure 4, C). Subsequently, a set of experiments was performed to find out the best media formulation to grow the cells for F_420_ production using an autoinduction protocol. ZY produced the highest F_420_ yield among ZY, YT, TB, SOB and SOC media. Bioinformatic analysis has indicated that FbiC is a protein with possible Fe–S clusters. In addition the reaction catalyzed by an archaeal homologue of FbiB requires L–glutamate and manganese chloride [22]. The expression media were therefore also supplemented with iron/sulphur and L–glutamate/manganese additives. The results indicated that supplementation of the expression media with either of these additives does indeed increase the F_420_ production yield (Figure 4, D). Surprisingly, cultures with an L–glutamate/manganese supplement did not have extra FO in the media, implying that the cells could convert all the produced FO into F_420_ inside the cells (Figure 4, D). It seems that the limiting factor in producing F_420_ from over–produced FO was the supply of the required L–glutamate/manganese.

**Figure 5:**
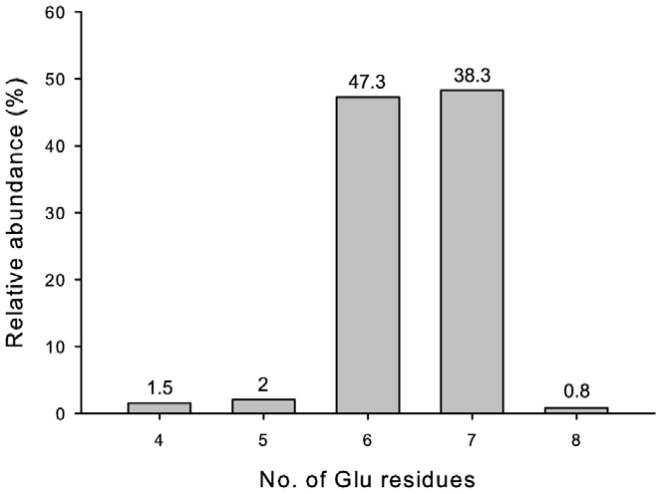
The F_420_ production profile from *M. smegmatis* cells over–expressing the FbiABC construct. The F_420_–6 and F_420_–7 species constitute the main species as deduced using mass spectrometry.

The FO/F_420_ produced by the cells expressing the FbiABC construct was purified and analysed using mass spectrometry. The results show two predominant fractions; a 200 mM NaCl fraction mainly composed of FO and a 500 mM NaCl fraction of exclusively F_420_ with more than 95% being F_420_-6 and F_420_-7 species (Figure 5). This result is in line with the previously published results of F_420_ extracted from the wild type *M. smegmatis* cells having the major species of F_420_-5 to F_420_-7 [9], [11], implying that the over–expression of the FbiABC construct does not change the F_420_ production profile.

## Discussion

The cofactor F_420_ has an important role in the metabolism of Archaea and has been the subject of numerous studies over the years since its identification. It is now clear that this importance applies also to Mycobacteria, for which there is growing evidence that F_420_ plays a key role in defence against oxidative and nitrosative stress [5], [24]. Consistent with this, the number of identified F_420_–dependent enzymes from Mycobacteria is growing, with nine new examples recently described [25]. A recent partial phylogenetic profiling study has proposed that there are at least 28 separate F_420_–dependent enzymes in *M. tuberculosis*, suggesting that F_420_ has a pivotal role in redox reactions of this pathogenic mycobacterium [26]. Few of these enzymes have been characterised, however, and research into their functions, and the role of F_420_, are handicapped by the fact that there is no commercial source for this cofactor, which can only be obtained in relatively low yield from the wild type *M. smegmatis* strain. A major aim of this study was to increase the F_420_ production yield in *M. smegmatis* by cloning and expression of the genes involved in F_420_ production and metabolism.

### Mycobacterial Expression Vectors

The pYUB1049 plasmid is a T7 promoter–based vector for which expression can be induced by IPTG or autoinduction. This vector has previously been used as a shuttle vector for cloning of Mycobacterial genes into *E. coli* and subsequent expression of proteins in *M. smegmatis* [17], [27]. The pYUB1049 vector has been also converted to a Gateway® cloning system compatible vector, pDESTsmg [18]. In this study, two different vectors were designed and prepared from the parental pYUB1049 vector; the pYUB28b vector is used for restriction/ligation cloning of single genes with the capability of expressing N– and C–terminal His–tags, whereas the pYUBDuet vector is a co–expression vector for simultaneous expression of two genes in a Mycobacterial host. Our experimental results demonstrate the application of T7–promoter based co–expression vectors in *M. smegmatis* that could also be useful in other contexts. Although there have been previous reports of co–expression systems for Mycobacteria [28], [29], [30], [31], [32], [33], to the best of our knowledge, this is the first T7–promoter based co–expression vector for a Mycobacterial host. The pYUB28b and pYUBDuet vectors, together with the pDESTsmg vector which has been previously developed in the authors' lab for the Gateway® cloning system, represent a repertoire of T7 promoter–based vectors which can be routinely used for expression of a wide range of ORFs in a Mycobacterial host.

### F_420_ Production

FbiC is annotated as FO synthase [34], catalysing the transfer of the hydroxybenzyl group from 4-hydroxyphenylpyruvate (a tyrosine precursor) to 5-amino-6-ribitylamino-2,4(1H,3H)-pyrimidinedione (an intermediate in flavin biosynthesis) to form FO (Figure 2) [23]. FO is the first intermediate with a complete deazaflavin chromophore in the F_420_ biosynthesis pathway [13], [23] providing the rationale for believing that this reaction might be the rate limiting step in F_420_ biosynthesis. FbiA and FbiB are believed to be involved in production of F_420_ from the precursor FO molecule; FbiA in generating F_420_–0 from FO and FbiB in adding glutamate residues to F_420_–0 to produce F_420_ with a poly–glutamate tail of variable length (Figure 2) [12]. In all Mycobacterial species with the genome sequences completed to date (21 in total, as of October 2010) FbiA is located immediately upstream of FbiB (www.TBdb.org). A detailed analysis indicates that the start site for the FbiB ORF overlaps with the last four base pairs of the FbiA ORF, though in a different reading frame, implying that they might be transcribed as a single operon for expression. In fact, it has been shown in *M. bovis* that these two ORFs are transcribed together as a single mRNA species [12]. This genetic arrangement made it possible to co–express the FbiAB operon and FbiC gene together, using the pYUBDuet vector, in order to increase F_420_ yield. Based on our results, the optimum condition to produce F_420_ by *M. smegmatis* cells expressing recombinant FbiABC is a culture with autoinduction media using ZY base over 4–5 days supplemented with iron, sulphur, L–glutamate and manganese. Using these optimal conditions, the F_420_ production yield was up to 10–times higher compared with the wild type strains.

The main limiting factor in F_420_ production, based on our results on over–expression of the three enzymes FbiA, FbiB and FbiC from the F_420_ biosynthetic pathway, appears to be the availability of the FbiB reaction substrate/cofactor. It does not seem that the FbiC reaction is the limiting step of the pathway even when the media are not supplemented with L–glutamate/manganese; excess FO was always present in high quantities in the media, indicating that the over–expressed FbiA and FbiB proteins are still not capable of converting all FO to F_420_. An alternative possibility is that FbiA and FbiB need other accessory protein(s) in order to perform the conversion more efficiently; in fact another ORF in *M. smegmatis* (MSMEG_2392) has been shown, by transposon mutagenesis studies, to be involved in F_420_ biosynthesis from FO [35]. Biochemical studies need to be performed using purified enzymes in order to study the kinetics in detail and determine the rate limiting step of the pathway.

Our previous crystal structures of *M. tuberculosis* FGD1 [9], together with other F_420_–containing crystal structures from different Archaeal species [36], [37], [38], [39], have indicated that the F_420_ poly–glutamate tail is not required for reaction catalysis; the poly–glutamate tail is extended into the solvent and it seems that this is a conserved feature of the enzymes that use F_420_ in oxidoreduction reactions. We propose that, therefore, the high yields of F_420_ from *M. smegmatis* strains expressing the recombinant FbiABC proteins, regardless of the number of glutamate residues in the poly–glutamate tail, identify this as a valuable source of F_420_ that might be used with enzymes purified from other microorganisms. Furthermore, the high yield of FO/F_420_ also opens a door for possible biotechnological applications.

### Depositions

The nucleotide sequences for pYUB28b and pYUBDuet vectors have been deposited in the National Centre for Biotechnology Information (NCBI) under GenBank HQ247814 and HQ247815 accession numbers, respectively. The vectors are available upon request.
